# Navigating Integration in Mixed‐Methods: A Practical Guide for Novice Nursing Researchers

**DOI:** 10.1002/nur.70048

**Published:** 2025-12-20

**Authors:** Natasha Hawkins, Sarah Yeun‐Sim Jeong, Jenny Sim

**Affiliations:** ^1^ School of Health University of New England Armidale New South Wales Australia; ^2^ School of Nursing, Faculty of Medicine and Health University of Sydney Camperdown New South Wales Australia; ^3^ NSW Head of School, School of Nursing, Midwifery & Paramedicine Australian Catholic University Sydney New South Wales Australia

**Keywords:** integration, methodology, mixed‐methods design, nursing research, sequential explanatory design

## Abstract

Mixed‐methods research is increasingly used in healthcare to understand complex issues, yet integration of qualitative and quantitative findings remains challenging, particularly for novice researchers. This paper provides a practical guide for nursing researchers to integrate survey and interview data effectively. Using a sequential explanatory design exemplar with an embedded quasi‐experimental component, we demonstrate key integration strategies, including joint displays and narrative weaving. The paper emphasizes that robust integration must be planned from the outset, with clear goals specifying how data types will address research questions. We illustrate how purposeful integration points, carefully constructed joint displays, and narrative synthesis of meta‐inferences can generate deeper insights than either method alone. By providing concrete examples and step‐by‐step guidance, this resource equips novice researchers with essential skills for achieving rigorous, cohesive integration in mixed‐methods studies.

## What Does This Paper Contribute to the Wider Global Clinical Community?

- The paper emphasizes that robust integration in mixed‐methods research must be planned from the outset. This involves setting clear goals that specify how qualitative and quantitative data will address the research questions, and applying structured approaches such as joint displays (tables, charts, or matrices) to align datasets. Presenting the final explanation in a logical and well‐organized way is critical, with narrative weaving–interlacing qualitative quotes and quantitative findings–offered as an effective strategy.
- By showcasing a sequential explanatory design with an embedded quasi‐experimental component, the paper provides a practical exemplar that equips novice researchers with the skills needed to achieve rigorous and cohesive integration in mixed‐methods studies.

## 1 Introduction

Mixed‐methods research (MMR) is an overarching research methodology that combines qualitative and quantitative methods in a single study. The collection, analysis, and purposeful integration of both quantitative (numerical) and qualitative (non‐numerical) data within a single study is a key characteristic of this methodology (Creswell and Clark 2007; Tashakkori and Teddlie 2003; Bazeley 2024). Purposeful integration enables researchers to draw on the complementary strengths of both approaches, addressing research questions more comprehensively than either method alone (Ivankova et al. 2006; Tashakkori and Teddlie 2003; Creswell and Clark 2018; Johnson et al. 2007). The added value of MMR is often summarized by the phrase “1 + 1 = 3,” reflecting the deeper insights that emerge when both forms of data are combined (Creswell 2024, p. 207).

Despite the growing prominence of MMR, particularly within the social, behavioral, and health sciences (O'Cathain et al. 2007; McKenna et al. 2021), there is still some disagreement about how MMR is defined (Kasirye 2021). A key distinction that requires clarity is between mixed‐methods research and multimethod research. MMR intentionally integrates both qualitative and quantitative approaches within a single study (Tashakkori and Teddlie 2003; Creswell and Clark 2018; Johnson et al. 2007). In contrast, multimethod research employs multiple data collection methods, which may include both qualitative and quantitative approaches, but crucially lacks the intentional integration of these data sets. In multimethod research, the different methods are used to address separate aspects of the research inquiry, with results typically reported separately rather than being systematically integrated (Kasirye 2021). While integration is crucial for MMR, representing the cornerstone that distinguishes it from other methodological approaches, researchers often find its effective execution challenging due to the complexity of meaningfully combining different types of data and maintaining methodological rigor throughout the integration process (Fetters et al. 2013; Cooper et al. 2024; McKenna et al. 2021).

Despite common misconceptions, integration is not a singular event that occurs at the end of a mixed‐methods study (Fetters et al. 2013; Yaqoob and Barolia 2023). Integration should be considered at the design, methods and interpretation and reporting phases of the study (Fetters et al. 2013). At the design level, integration can be incorporated into a plan for how quantitative and qualitative components will align, such as through exploratory‐sequential, explanatory‐sequential, or convergent designs (Fetters et al. 2013; Creswell and Clark 2007; Yaqoob and Barolia 2023). At the methods level, data from different approaches are systematically linked using techniques like connecting (linking data through sampling), building (one dataset informing another), merging (combining datasets for examination), or embedding (frequently linking data collection and analysis) (Yaqoob and Barolia 2023). Finally, integration extends to the interpretation and reporting level, where findings are synthesized through narrative approaches, data transformation (e.g., quantifying qualitative data), or visual tools like joint displays to form a holistic understanding (Yaqoob and Barolia 2023; Cooper et al. 2024). While integration is crucial for MMR, its effective execution remains a common challenge in practice, often being underdeveloped or underutilised (Fàbregues et al. 2022; Morgan 2023; Younas and Durante 2023).

This lack of integration can result in fragmented findings, missed insights, and a failure to fully capitalize on the strengths of the mixed‐methods approach (Cooper et al. 2024; Fàbregues et al. 2022). The challenges associated with integration are multifaceted, encompassing conceptual, practical, methodological, and reporting dimensions (Oranga 2025; McKenna et al. 2021). These difficulties are particularly pronounced for novice researchers, who may find navigating the complexities of integrating quantitative and qualitative components daunting. Furthermore, there has been a recognized shortage of detailed, well‐articulated techniques and practical exemplars demonstrating *how* to implement integration effectively throughout all stages of a study (Oranga 2025).

Effectively implementing integration procedures in MMR requires a diverse skill set, including competence in both qualitative and quantitative approaches, something that can be particularly challenging for novice researchers. Although frameworks such as the Mixed‐Methods Integration Quality Framework (MMIQF) (Fàbregues et al. 2024) offer valuable guidance for designing and reporting integration, their practical application is still underrepresented in the literature. In addition, while recent contributions, such as those by Cooper et al. (2024), have expanded the definition of data sources suitable for integration to include elements like rigorous literature reviews, there remains a critical need for concrete, step‐by‐step examples of how to integrate traditional qualitative and quantitative data in real‐world studies. To ensure transparency and rigor in addressing this gap, this paper adheres to the Good Reporting of A Mixed‐Methods Study (GRAMMS) guidelines (O'cathain et al. 2008), which provide a structured framework for comprehensive reporting of MMR. By demonstrating adherence to GRAMMS, we complement the integration techniques discussed with clear communication of both the process and outcomes of integration (Supporting Information S1).

This paper addresses the need for practical guidance by offering a transparent and practical model for navigating integration across all stages of a mixed‐methods study. Using an exemplar from a study (Hawkins 2025) that employed a sequential explanatory design with an embedded quasi‐experimental component, we aim to demonstrate how integration can be meaningfully planned, implemented, and reported. The purpose of this paper is to provide a resource for novice nursing researchers seeking to enhance the rigor and utility of their mixed‐methods work.

## 2 Overview of the Exemplar Study

The exemplar study employed a sequential explanatory design with an embedded quasi‐experimental component. This design entailed collecting and analyzing quantitative data first, followed by the collection of qualitative data to provide further context and understanding of the initial quantitative results. The quasi‐experimental component involved the implementation of an intervention, with a control group for comparison. The study protocol, detailing the research aims, methodology, and analyses, has been published previously (Hawkins et al. 2021a). Ethical approval for the study was obtained from the Local Health Districts Human Research Ethics Committee (HREC) (NSW HREC Reference No: HREC/17/HNE/596).

### 2.1 Theoretical Framework

Social Worlds Theory (Clarke 1991) provided the theoretical framework that underpinned the mixed‐methods study. This theory conceptualizes society as interconnected social worlds, where individuals simultaneously belong to and construct multiple social worlds through repeated interactions (Carter and Fuller 2015; Strauss 1978). Each social world is characterized by shared activities, communication networks and specific sites of operation (Strauss 1978). This theoretical framework inherently justifies the need for both quantitative and qualitative approaches, as neither method alone can fully capture how people move between and exist in different professional spaces. The integration of both approaches allows us to examine the measurable structural elements while simultaneously exploring the rich interpretive dimensions of social phenomena. We leveraged this framework to guide our integration of quantitative data about observable activities with qualitative insights into authenticity and belonging, inform our analysis of power relationships and conflicts (Strauss 1982), and frame our understanding of how individuals navigate between different social worlds (Strauss 1978). This theoretical lens enhanced our integration process by providing a coherent framework for combining structural measurements with rich descriptions of lived experiences within and between social worlds.

### 2.2 Aim

The study aimed to investigate the self‐reported exposure to and experiences of negative workplace behaviors of nursing staff and their ways of coping in regional acute care hospitals in one local health district (LHD) in NSW before and after the intervention “Respectful Workplace Workshops” were implemented within the organization.

### 2.3 Data Collection

Data collection occurred in two distinct phases, each serving a specific purpose in understanding the intervention's effectiveness.

- **Quantitative Phase:** Surveys were administered as pre‐ and post‐test measures to assess the intervention's impact on the levels and types of negative workplace behavior experienced by participants. These initial findings identified key patterns and areas requiring deeper investigation.
- **Qualitative Phase:** Building on the quantitative results, semi‐structured interviews were then conducted to explore why and how these changes occurred, capturing the nuanced experiences and contextual factors that numerical data alone could not reveal.

This sequential design was chosen deliberately—the quantitative phase provided a broad overview of the intervention outcomes, while the subsequent qualitative phase allowed us to not only explore the effects of the intervention, but also to provide insights into the complex social dynamics and personal experiences of participants. This was something that quantitative measures alone could not fully capture.

## 3 Integration Procedure

The research team recognized that integration across design, analysis, and interpretation phases was crucial for leveraging the full potential of mixed‐methods research (MMR) and achieving optimal research outcomes (O'Cathain et al. 2007). This strategic approach was designed to generate comprehensive insights that could effectively inform both practice and policy development. The following discussion examines how strategic integration planning enhanced the study's outcomes.

### 3.1 Strategic Integration Planning

The foundation of successful mixed‐methods research lies in early integration planning (Fetters 2019). A well‐structured approach ensures coherence across data collection, analysis, and interpretation phases. Early‐stage integration planning creates alignment between qualitative and quantitative elements, ensuring methodological compatibility and complementarity in addressing research objectives (Fetters et al. 2013). This proactive planning helps prevent fragmented findings by establishing clear integration objectives from the outset (O'Cathain et al. 2007; Fetters et al. 2013).

Following the approach of Fetters et al. (2013), the research team, equipped with the necessary skills (proficiency in both qualitative and quantitative methods), time, and resources, began the study with clearly defined research objectives that outlined how the quantitative and qualitative components would each contribute to addressing the research questions. The team implemented a sequential explanatory design, where quantitative data collection and analysis preceded qualitative investigation, allowing the qualitative phase to elaborate on and explain quantitative findings (Ivankova et al. 2006). A comprehensive study protocol, including a detailed mixed‐methods procedural diagram, was developed and published to outline the integration timeline and methodology (Hawkins et al. 2021a). Integration was structured through strategic connection points between datasets, including participant sampling strategies and the sequential building of qualitative inquiry upon quantitative findings (Fetters et al. 2013).

To ensure methodological coherence, the team developed clear alignments between datasets and research questions. A visual representation was created to illustrate these connections (Plano Clark 2019), demonstrating how each research question would be addressed and highlighting the ways qualitative data would expand upon survey findings (See Table 1).

**Table 1:** Alignment of data sources to research questions (from Hawkins 2025).

Research question	Survey items	Interview questions
What are the current level and types of negative workplace behaviors occurring among nurses in regional acute care settings? What are the current level and types of negative workplace behaviors occurring towards specifically new graduate nurses in regional acute care settings?	Q36–41 Have you been bullied at work over the past one month? If yes, who were you bullied by (role/gender)? Have you experienced incivility over the past one month? If yes who was uncivil (role/gender)? Part 2 ‐ Negative Acts Questionnaire – Revised (NAQ‐R) (Einarsen et al. 2009)	*What can you tell me about the organizational culture in your current workplace?* *Have you ever experienced or observed negative workplace behavior in your current workplace?* *How do you feel about other members of the nursing profession support towards new graduates?* *Do you think that new graduate nurses are more likely to be the victims of bullying and incivility?* *What negative behaviors have you experienced or observed your workplace?* *Can you give me an example of bullying behaviors you have experienced?* *Can you give me an example of incivility you have experienced?*
What are the characteristics of respectful workplace behaviors that can be promoted?	Not applicable	*What positive behaviors have you experienced or observed in your workplace?*
How do nurses cope when exposed to negative workplace behavior?	Turnover intentionPart 3 – Ways of Coping Questionnaire (revised) (Folkman and Lazarus 1985)	*Do you feel accepted and like you belong as a member of the nursing profession?* *Do you agree with the statement that all nurses are accepted and feel like they belong as a member of the nursing profession?* *Tell me about how you dealt with negative workplace behavior?* *Do you feel that this negative behavior had any effect upon your work?* *Do you feel like this impacted upon patient care you deliver?*
Do the Respectful Workplace Workshops have an impact on the ways nurses cope when exposed to negative workplace behavior? What are the effects of Respectful Workplace Workshops on nurses’ knowledge and skills to respond to negative behavior?	pre‐ and post‐Intervention Q36–41 Have you been bullied at work over the past one month? If yes, who were you bullied by (role/gender)? Have you experienced incivility over the past one month? If yes who was uncivil (role/gender)? pre‐ and post‐Intervention Part 2 ‐ Negative Acts Questionnaire – Revised (NAQ‐R) pre‐ and post‐Intervention Q43 Response to bullying questions (Likert scale)	*Could you please describe how you felt about the Respectful Workplace Workshops?* *Do you feel that the workshops were beneficial?* *What do you know about the NSW Health policy on Bullying and Incivility?* *What do you understand bullying to be?* *What do you understand incivility to be?* *Do you feel that the workshops were beneficial?* *After attending the Respectful Workplace Workshops what changes (if any) did, or will you implement?* *After attending the Respectful Workplace Workshops what changes (if any) did, or will you implement?* *What changes (if any) have you observed in the wards you have been working?*

Abbreviation: NAQ‐R, Negative Acts Questionnaire Revised.

### 3.2 Interpretation: Creating Joint Displays

At the interpretation and reporting level, integration was planned to occur through joint displays and a weaving narrative approach (Fetters et al. 2013). Joint displays “bring the data together through a visual means to draw out new insights beyond the information gained from the separate quantitative and qualitative results” (Fetters et al. 2013, p. 2143). The most common type of joint display is a side‐by side display that juxtaposes qualitative results with related quantitative results and resulting meta inferences (Guetterman et al. 2021). Throughout this process, the research team examined findings for confirmation (qualitative data validating quantitative findings), expansion (findings from both datasets broadening understanding by examining different aspects), and discordance (inconsistent or contradictory results) (Fetters et al. 2013).

According to Fetters (2019), a joint display comprises a table or figure that organizes and analyses mixed‐methods research data. Such displays align qualitative and quantitative findings side‐by‐side, emphasizing connections and shared themes. The joint display not only visually represents these linkages, which can be expressed as constructs or domains, but also includes an interpretation of what the combined results mean, often referred to as “meta‐inferences.” Essentially, it provides a visual and interpretive framework for understanding the interplay of qualitative and quantitative data (Fetters et al. 2013; Guetterman et al. 2021).

The research team selected joint displays for integration because of their capacity to generate new understanding as researchers simultaneously consider quantitative and qualitative components (Fetters and Tajima 2022; Haynes‐Brown and Fetters 2021). Haynes‐Brown and Fetters (2021, p. 3) describe the development of joint displays as “flexible and iterative,” typically involving multiple revisions towards the final presentation (Guetterman et al. 2021). The iterative development process forms a crucial part of analysis (Fetters and Tajima 2022; Haynes‐Brown and Fetters 2021). In developing data integration for this study, the research team created multiple iterations of joint displays to identify correlations between datasets (Fetters and Tajima 2022). While recent literature has begun documenting this process (Fetters and Tajima 2022; Guetterman et al. 2021), detailed examples of iterative joint display construction remain limited (Haynes‐Brown and Fetters 2021). Our team's initial display comprised a simple two‐column table headed “Quantitative Results” and “Qualitative Results” (See Supporting Information S2). The team entered primary findings from both research strands for side‐by‐side comparison, then systematically analyzed both datasets to identify areas of convergence and divergence.

The team then grouped key findings from both datasets by related concepts within the joint display. This process revealed interrelated concepts, leading to the development of overarching themes to demonstrate these relationships. These themes were incorporated into the next iteration under “Themes” and “Concepts” headings. Following this, the team undertook “fit” labeling, comparing data for confirmation, expansion or discordance (Fetters et al. 2013). Confirmation labels indicated qualitative data supporting quantitative findings; expansion labels showed qualitative data providing additional insights; and discordance labels identified inconsistent or contradictory findings between datasets (Fetters et al. 2013). An excerpt from the final joint display (See Supporting Information S3), is presented in Table 2 and shows quantitative and qualitative data side‐by‐side, demonstrating “fit” through confirmation, expansion and discordance (Fetters et al. 2013). This display illustrates how key findings align with developed concepts and themes, which informed meta‐inference exploration through narrative synthesis.

**Table 2:** Theme 1—Joint Display (From Hawkins 2025).

Theme	Concept	Quantitative data key findings	Qualitative data key findings	Meta‐inferences
*Workplace behaviors within the Nursing Social World*	**Everyone is at risk of negative behavior exposure.**	Across the total sample (*n* = 130), 31% (*n* = 40) experienced bullying, and 46% (*n* = 59) experienced incivility. There was no significant difference in nursing roles experiencing negative behaviors. 48% (*n* = 12) of participants identified managers as the main perpetrators of bullying in the pre‐intervention survey. Registered Nurse (RN) colleagues were identified as being the main source of incivility (42%, *n* = 15) in the pre‐intervention data and in the post‐intervention data (57%, *n* = 13).	All informants at all occupational levels experienced negative workplace behaviors. Informants described RN colleagues and managers as being the perpetrators of negative workplace behaviors. Informants described how wards were like “tribes” and that there were barriers to the acceptance of individuals into that tribe. These barriers included a nurse's way of working and how they practice, and certain personal attributes. Informants described how any new staff to a ward were more likely to be the victims as perpetrators saw them as being vulnerable and weak.	***Confirmation*** The qualitative data confirm the presence of negative workplace behaviors within the nursing social world. It occurs at all levels of nursing, and RN colleagues and managers were identified as being the main source of negative workplace behaviors. ***Expansion*** Insights gained from the qualitative component identified territorial, tribal behaviors that impacted socialization in the nursing social world. The ability to achieve and adhere to the in‐world standards of practice was also identified as being a reason that nurses would be exposed to negative behaviors.
*Workplace behaviors within the Nursing Social World*	**It's not just personal acts; it's work‐related acts facilitated by organizational factors.**	The most reported negative acts experienced by participants across all sites in both surveys were *work‐related bullying acts* and included being exposed to an unmanageable workload (75%, *n* = 97), having opinions and views ignored (49%, *n* = 64), being given tasks with unreasonable targets or deadlines (48% *n* = 63), someone withholding information that affects your performance (44% *n* = 57).	The behaviors that informants were exposed to in their workplace included unfair rostering and workloads, made to work outside of their skill level, isolation and exclusion, information being withheld, being undermined and contradicted in front of the team and being singled out and made to look incompetent. Informants described how negative behavior was often tolerated and normalized. They suggested that the zero‐tolerance policy was a joke and not actually reinforced. Informants reported that poor workplace conditions, such as short staffing and heavy workloads, negatively affected nurses’ workplace interactions. Nurse Unit Manager (NUM) 3 described negative workplace behaviors as “a symptom of a struggling system.” The ward was described as a reflection of the NUM's leadership. Informants suggest that senior management was disconnected from the front line and was focused only on meeting their KPIs.	***Confirmation*** Both the quantitative and qualitative data identify work‐related bullying acts, in particular workload allocation, as the most experienced negative act. ***Expansion*** The qualitative data also identified person‐related acts such as isolation and exclusion and being singled out in front of the team. The qualitative data provided insights into a struggling health care system where poor workplace conditions, leadership voids, a lack of management of negative behaviors and a perceived disconnect from management were suggested to intensify negative workplace behaviors further.

### 3.3 Presenting the Narrative

The integration process progressed to developing a narrative synthesis of meta‐inferences. Following Fetters et al.‘s (2013) “weaving approach,” the research team structured the presentation around themes and concepts identified in Table 2. Each theme and its associated concepts were systematically explored, weaving together both data strands. The following excerpt from Hawkins (2025) illustrates this approach through the first theme of the narrative synthesis, where qualitative and quantitative findings from the exemplar study are integrated.

### 3.4 Narrative Synthesis

#### 3.4.1 Theme 1: Workplace Interactions Within the Nursing Social World

This concept explores workplace interactions within the nursing social world and outlines the prevalence of negative workplace exposure and the positive workplace practices that should be promoted to assist with the creation of a respectful workplace. The data demonstrate that negative behaviors are not just personal acts but are often work‐related bullying acts that can be facilitated by certain organizational factors.

#### 3.4.2 Everyone Is at Risk of Negative Behavior Exposure

Data from the quantitative strand phase clearly identifies the occurrence of negative workplace behaviors across the total sample (*n* = 130), with 31% of participants (*n* = 40) experiencing bullying and 46% of participants (*n* = 59) experiencing incivility (Hawkins et al. 2023a). Although new graduate RNs have been identified as being at greater risk of negative behaviors in other studies (Weaver 2013), there was no significant difference between the nursing roles and their experience of negative behaviors in this study (Hawkins et al. 2021b). All nursing informants from the qualitative phase (including new graduate nurses, RNs, clinical nurse educators and NUMs) also identified that they had experienced negative workplace behaviors within the nursing profession (Hawkins et al. 2021b). The nurse informants identified that it was any new staff of any level to a ward who were more likely to be victims as they were seen as being vulnerable and weak (Hawkins et al. 2023a; Hawkins et al. 2021b).

As previously reported in other studies, power and hierarchy are the main features of negative workplace behavior (Hawkins et al. 2023b). It is also commonly reported that the perpetrators of negative behaviors are people with more “power” (Hawkins et al. 2023b). This study found that NUMs and RN colleagues were the main perpetrators of bullying and incivility in both the pre‐ and post‐intervention surveys (Hawkins et al. 2021b; Hawkins et al. 2023a). In the pre‐intervention survey, 48% (*n* = 12) of participants identified managers as the main perpetrators of bullying; however, in the post‐intervention period, 73% (*n* = 11) of participants identified RN colleagues as the main perpetrators. (Hawkins et al. 2021b). RN colleagues were identified as being the main source of incivility in both the pre‐ (42%, *n* = 15) and post‐intervention (57%, *n* = 13) surveys. The nurse informants also described their experiences of negative workplace behaviors and identified both colleagues and managers (NUMs and senior management) as the perpetrators (Hawkins et al. 2023a).

The qualitative strand allowed for an in‐depth exploration of the workplace behaviors occurring within the nursing social world. It provided context and identified contributing factors for negative workplace behaviors. Insights gained from the qualitative component identified territorial, tribal behaviors that impacted socialization in the nursing social world, with participants describing their tribes as having an “us versus them” (Clinical nurse educator 1) attitude and “being like a pack” (Clinical nurse educator 6) (Hawkins et al. 2023b). The ability to achieve and adhere to the in‐world standards of practice was also identified as a reason nurses would be exposed to negative behaviors. Nurses were expected to do “what has always been done” and not question practice (Hawkins et al. 2023b). Segmentation exists within the nursing social world, with more experienced nurses identified as “authenticators,” deciding who belongs on the ward. The ability to complete the required workloads and adhere to the wards’ “ways of working” was identified as a major benchmark to achieving acceptance into the social world (Hawkins et al. 2023b). Nurses described having to prove themselves before being accepted, with some reporting that they remained as outcasts and were still searching for their “niche” where they might belong (Hawkins et al. 2023b).

#### 3.4.3 It's Not Just Personal Acts; It's Work‐Related Acts Facilitated by Organizational Factors

Both the quantitative and qualitative data sets identified work‐related bullying acts, in particular, workload allocation, as the most commonly experienced negative act (Hawkins et al. 2023a; Hawkins et al. 2021b; Hawkins et al. 2023b). The behaviors that informants identified during the interviews included unfair rostering and workloads, being made to work outside of their skill level, isolation and exclusion, information being withheld, being undermined and contradicted in front of the team and being singled out and made to look incompetent (Hawkins et al. 2023b). The qualitative data also provided insights into a struggling health care system where poor workplace conditions, leadership voids, a lack of management of negative behaviors and a perceived disconnect from management were suggested to intensify negative workplace behaviors further (Hawkins et al. 2023b).

The synthesis of quantitative prevalence statistics with detailed qualitative accounts reveals that negative workplace interactions are not isolated events, but rather manifestations of deeper cultural and structural patterns. By combining both datasets, a more sophisticated and thorough understanding emerges, where quantitative findings establish magnitude and frequency, while qualitative insights provide contextual depth and meaningful interpretation. This methodological strength enhances the capacity for researchers, policymakers, and healthcare leaders to develop evidence‐informed initiatives and guidelines that are both specific and situationally appropriate. Such integrated understanding ultimately facilitates the creation of comprehensive, workable approaches to address negative behaviors while fostering a professional culture built on respect and inclusivity.

## 4 Lessons Learned and Practical Tips for Novice Researchers

The study described in this paper emerged from doctoral research (Hawkins 2025) completed in 2023 and serves as an exemplar for novice nursing researchers navigating the complexities of mixed‐methods integration. While many researchers understand the importance of collecting both quantitative and qualitative data, effective integration requires careful consideration from the earliest stages of study design through to final analysis and reporting.

Integration must begin at the conceptual phase, with researchers clearly articulating how each data type will contribute to addressing specific research questions (Fetters et al. 2013; Yaqoob and Barolia 2023). This involves more than simply collecting different types of data; it requires strategic planning of integration points where datasets will inform and enhance each other. For example, in this study, quantitative findings shaped the development of interview questions, allowing for deeper exploration of emerging patterns.

The successful execution of integration relies heavily on appropriate analytical tools and presentation strategies. Joint displays—including tables, matrices, and visual representations—serve as crucial bridges between datasets, enabling researchers to identify patterns, convergences, and divergences (Fetters et al. 2013; Cooper et al. 2024; Guetterman et al. 2021). These tools facilitate systematic comparison and synthesis of findings, moving beyond parallel presentation to true integration.

In presenting integrated findings, narrative weaving emerged as a particularly effective strategy. This approach interweaves quantitative results with qualitative insights, creating a coherent narrative that demonstrates how different data types complement and enhance understanding. Importantly, when datasets diverge, these differences should not be viewed as problematic but rather as opportunities for deeper insight into the complexity of the phenomenon under study (Fetters et al. 2013).

Finally, beyond integration techniques, novice researchers should familiarize themselves with established reporting standards such as the Good Reporting of A Mixed‐Methods Study (GRAMMS) checklist (O'cathain et al. 2008), which ensures comprehensive documentation of mixed‐methods procedures and findings.

## 5 Implications for Nursing Research

Implementing integration in mixed‐methods research is not a linear process. Integration requires thoughtful reflection, methodological rigor, and a commitment to honoring the contributions of both qualitative and quantitative strands (Haynes‐Brown and Fetters 2021). This paper has provided a practical, experience‐based guide to help novice researchers approach integration with greater confidence. By using a sequential explanatory design with an embedded quasi‐experimental component, the exemplar study demonstrated how purposeful design, alignment with research questions, and early planning for integration can yield cohesive and meaningful results.

For novice researchers, the integration phase can initially seem daunting. However, as illustrated through this guided approach, integration becomes more manageable when viewed as a process of sense‐making, bringing together different forms of evidence to tell a more complete story. It is in this phase that the real strength of mixed‐methods research emerges, offering deeper insights than either method could provide alone.

Ultimately, integration is not only a technical task but also a conceptual one. Integration requires researchers to remain grounded in their research purpose, transparent in their methods, and open to the dynamic interplay between numbers and narratives (Fetters et al. 2013; Haynes‐Brown and Fetters 2021). As the field of nursing continues to embrace complexity in research techniques, the ability to integrate findings effectively will remain a vital skill. We hope that the strategies and reflections shared in this paper will support emerging researchers in developing this skill, contributing to the growth of high‐quality, mixed‐methods nursing research.

## 6 Conclusion

Robust integration of quantitative and qualitative data in mixed‐methods research (MMR) is fundamental to advancing nursing knowledge, particularly given the complexity of healthcare environments where measurable outcomes intersect with human experiences. This paper demonstrates how purposeful integration of survey data and individual interviews can generate deeper insights than either method alone. Through planned integration points, carefully constructed joint displays, and narrative synthesis of meta‐inferences, we illustrate how different data types can work synergistically to create a more comprehensive understanding of complex nursing phenomena.

However, effective integration demands more than simply collecting both types of data. Researchers must develop sophisticated methodological expertise to navigate these complexities successfully. This includes not only proficiency in both qualitative and quantitative approaches but also mastery of specific integration tools and techniques. Without these skills, researchers risk producing parallel findings that fail to harness the full potential of MMR. The integration process demonstrated in this paper provides novice researchers with practical strategies for meaningful data synthesis, moving beyond basic combination to true integration that can inform evidence‐based practice and healthcare policy.

This methodological expertise is particularly crucial in nursing research, where understanding both the measurable outcomes and the lived experiences of healthcare delivery is essential for advancing practice. By providing a detailed exemplar of successful integration, this paper contributes to building capacity in mixed‐methods expertise among nursing researchers.

## Author Contributions

**Natasha Hawkins:** conceptualization, methodology, analysis, writing – original draft, review and editing. **Sarah Yeun‐Sim Jeong:** supervision, methodology, analysis, writing – review and editing. **Jenny Sim:** supervision, methodology, analysis, writing – review and editing.

## Funding

The authors received no specific funding for this work.

## Conflicts of Interest

The authors declare no conflicts of interest.

## Supporting information


**Supplementary File 1:** GRAMMS Checklist.

**Supplementary File 3:** ‐ Final Joint Display.

**Supplementary Table 2:** Initial Joint Display FINAL.

## Data Availability

The data that support the findings of this study are available in the supplementary information of this article.
