# Supplementary material for: Navigating Integration in Mixed‐Methods: A Practical Guide for Novice Nursing Researchers
Source: Res Nurs Health. 2025 Dec 20;49(2):193–201. doi: 10.1002/nur.70048 (PMC12954644; doi:10.1002/nur.70048)
Supplement: Supplementary file 1 — GRAMMS Checklist. [file NUR-49-193-s001.docx]

| **Guideline** | **Section: page** |
| --- | --- |
| ***Describe the justification for using a mixed methods approach to the research question*** | Theoretical Framework: page 5 (paragraph explaining how Social Worlds Theory "inherently justifies the need for both quantitative and qualitative approaches") |
| ***Describe the design in terms of the purpose, priority and sequence of methods*** | Overview of the Exemplar Study: page 5 (sequential explanatory design with embedded quasi-experimental component); Data Collection: page 6 (describes two distinct phases and their purposes) |
| ***Describe each method in terms of sampling, data collection and analysis*** | Data Collection: page 6 (describes quantitative surveys and qualitative semi-structured interviews); Strategic Integration Planning: page 7 (mentions sampling strategies); Table 1: page 8 (alignment of survey items and interview questions) |
| ***Describe where integration has occurred, how it has occurred and who has participated in it*** | Integration Procedure: pages 6-7; Strategic Integration Planning: page 7 (connection points between datasets); Interpretation: Creating joint displays: pages 8-10; Presenting the narrative: pages 10-13 |
| ***Describe any limitation of one method associated with the present of the other method*** | Lessons Learned and Practical Tips: page 13 (discusses divergent datasets as opportunities); Conclusion: page 14 (mentions risks of producing parallel findings without proper integration skills) |
| ***Describe any insights gained from mixing or integrating methods*** | Narrative Synthesis: pages 11-13 (demonstrates insights from integration); Theme 1 example: pages 11-13 (shows how integration revealed "deeper cultural and structural patterns"); Conclusion: page 14 ("generate deeper insights than either method alone") |

*Supplementary Table 1: GRAMMS Checklist*

O'Cathain A, Murphy E, Nicholl J. The quality of mixed methods studies in health services research. J Health Serv Res Policy. 2008;13: 92-98
