# Supplementary material for: Navigating Integration in Mixed‐Methods: A Practical Guide for Novice Nursing Researchers
Source: Res Nurs Health. 2025 Dec 20;49(2):193–201. doi: 10.1002/nur.70048 (PMC12954644; doi:10.1002/nur.70048)
Supplement: Supplementary file 2 — Supplementary File 3: ‐ Final Joint Display. [file NUR-49-193-s002.docx]

*Supplementary 3: Joint Display (From BLINDED FOR REVIEW, 2025)*

| Theme | | Concept | Quantitative Data Key Findings | | | Qualitative Data Key Findings | Meta-inferences | |
| --- | --- | --- | --- | --- | --- | --- | --- | --- |
| *Workplace behaviours within*  *the Nursing Social World* | | **Everyone is at risk of negative behaviour exposure.** | Across the total sample (n = 130) 31% of participants (n = 40) experienced bullying, and 46% of participants (n = 59) experienced incivility.  There was no significant difference in nursing roles experiencing negative behaviours.  48% (n = 12) of participants identified managers as the main perpetrators of bullying in pre-intervention survey.  RN colleagues were identified as being the main source of incivility (42%, n = 15) in the pre-intervention data and in the post-intervention data (57%, n = 13). | | | All informants at all occupational levels experienced negative workplace behaviours.  Informants described RN colleagues and managers as being the perpetrators of negative workplace behaviours.  Informants described how wards were like ‘tribes’ and that there were barriers to the acceptance of individuals into that tribe.  These barriers included a nurse’s way of working and how they practice, and certain personal attributes.  Informants described how any new staff to a ward were more likely to be the victims as perpetrators saw them as being vulnerable and weak. | ***Confirmation***  The qualitative data confirm the presence of negative workplace behaviours within the nursing social world. It occurs at all levels of nursing, and RN colleagues and managers were identified as being the main source of negative workplace behaviours.  ***Expansion***  Insights gained from the qualitative component identified territorial, tribal behaviours that impacted socialisation in the nursing social world.  The ability to achieve and adhere to the in-world standards of practice was also identified as being a reason that nurses would be exposed to negative behaviours. | |
| *Workplace behaviours within*  *the Nursing Social World* | | **It’s not just personal acts; it’s work-related acts facilitated by organisational factors.** | The most reported negative acts experienced by participants across all sites in both surveys were *work-related bullying acts* and included being exposed to an unmanageable workload (75%, n = 97), having opinions and views ignored (49%, n = 64), being given tasks with unreasonable targets or deadlines (48% n = 63), someone withholding information that affects your performance (44% n = 57). | | | The behaviours that informants were exposed to in their workplace included unfair rostering and workloads, being made to work outside of their skill level, isolation and exclusion, information being withheld, being undermined and contradicted in front of the team and being singled out and made to look incompetent.  Informants described how negative behaviour was often tolerated and normalised. They suggested that zero-tolerance policy was a joke and not actually reinforced.  Informants identified that poor workplace conditions, such as short staffing and heavy workloads, had negative effects on nurses’ workplace interactions.  NUM3 described negative workplace behaviours as ‘a symptom of a struggling system’.  The ward was described as being a reflection of the NUM’s leadership.  Informants suggest that senior management was disconnected from the front line and was focused only on meeting their KPIs. | ***Confirmation***  Both the quantitative and qualitative data identify work-related bullying acts, in particular workload allocation, as the most experienced negative act.  ***Expansion***  The qualitative data also identified person-related acts such as isolation and exclusion and being singled out in front of the team.  The qualitative data provided insights into a struggling health care system where poor workplace conditions, leadership voids, a lack of management of negative behaviours and a perceived disconnect from management were suggested to intensify negative workplace behaviours further. | |
|  |  | **There are some positive behaviours to be promoted.** | No data | | | Informants spoke about instances of tiny individual acts of kindness and positive workplace practices.  The acceptance of learning as a lifelong process was also viewed as a crucial underpinning to positive workplace culture.  When staff spoke about organisational commitment to promoting positive behaviour, they suggested that having more staff would improve workplace culture.  Informants also spoke about feeling offended by organisational attempts to ‘reward’ staff with token gestures such as ‘bacon and egg rolls’. | ***Expansion***  The quantitative survey responses did not allow for exploration of the types of positive workplace behaviours that could be promoted to create respectful workplace conditions. However, the qualitative data allowed for exploration of these ‘tiny acts of kindness’ and the positive impact they had on nurses. | |
| Impacts of negative workplace *behaviours* | Nurses are leaving. | | | 51% (n = 21) of participants in the pre-intervention and 48% (n = 12) of participants in the post-intervention indicated they had thought about leaving their current position due to negative behaviours experienced. | Negative behaviour was cited by informants as a reason that nurses leave the profession and had implications for recruitment. | | **Confirmation**  Both the quantitative and qualitative data identify negative behaviours as a threat to the recruitment and retention of nurses in workplaces. | |
|  | **It negatively impacts not only**  **the victim but also the**  **organisation and risks patient safety.** | | | No data | One informant described it as a ‘living hell’ (NUM1), and others shared feelings of embarrassment, fear, sadness, tiredness and feeling physically sick. They describe lacking self-confidence and the motivation to come to work, feeling burnt out and considering leaving, with new graduates questioning their career choice.  The behaviours impacted informants’ home lives with reports of increased irritability at home, increased alcohol consumption and lack of sleep due to thinking about incidents that had occurred.  Informants also reported how behaviour affected the wards’ culture and working environment and had implications for patient safety and the quality of care they received. | | **Expansion**  The qualitative data provided insight into impacts at a personal level that highlight the profound effects that negative behaviours can have upon individuals. This included insights into the impact on life and health outside of work.  One aspect often overlooked is the consequence of the quality of patient care. This was explored in the qualitative data. | |
|  | **Nurses cope with exposure in a variety of ways.** | | | The most common ways of coping used by participants in both the pre- and post-intervention data included concentrating on what they had to do next (91%, n = 113), trying to analyse the problem to understand it better (90%, n = 110), turning to a work or substitute activity to take their mind off it (80%, n = 98) and talking to someone about how they were feeling (79%, n = 93).  The most used coping methods were in Domain 1 - Problem-focused coping, followed by Domain 4 - Seeking social support.  Although reported in the bottom five ways of coping, 26% (n = 18) of participants in the pre-intervention survey and 23% (n = 27) of participants in the post-intervention survey reported ‘taking it out on other people’. | Informants reported using various ways of coping, ranging from exercising, avoidance of the person/workplace, keeping work and life separate, giving up and acquiescing to fit in, or formulating a plan to deal with the negative behaviour.  Informants also reported seeking support from others, including family, mentors and from Employee Assistance Program (EAPs). Some informants reported taking sick leave or considered leaving their job.  Retaliation became a mechanism for coping. Informants reported that in response to negative behaviour experienced, they began to withhold information, do the bare minimum and refuse to help others. | | **Confirmation**  Both the quantitative and qualitative data sets identified problem-focused coping strategies implemented by participants to try to alter their situation. They both also explored the important strategy of seeking support from others.  **Expansion**  The qualitative data identified that supportive people could include family members, mentors and EAPs.  The qualitative data also explored specific negative coping responses used by informants. | |
| *Intervention evaluation* | **Small improvements and initial inspiration.** | | | There was a decrease in reported bullying in both the intervention group and control group, from 37% to 31% and from 29% to 21%, respectively.  There was a decrease in the number of participants who experienced incivility in the control group from 57% to 26%; however, the intervention group reported a rise from 44% to 50%.  For the NAQ-R Domain 1 - Work-related bullying exposure, the intervention group had a median score change of –2 (95% CI = –5.5 to 1.5) from the pre-intervention period.  For the intervention group, there were statistically significant decreases in the Ways of Coping Questionnaire Domain 4 – Seeking support (p = 0.019) and Domain 6 – Self-blame (p = 0.011) as coping strategies after exposure to the Respectful Workplace Workshops.  Improvement in the intervention groups’ policy awareness from 82% (n = 37) to 92% (n = 33).  Improvements were seen in intervention participants’ perceived ability to challenge disrespectful behaviour, ability to use the resolution pathways and knowing when to escalate to management, although these changes were not statistically significant. | Informants described a ‘big push’ to attend the educational intervention and that staff initially had inspiration after first attending the workshops, as they were ‘all on the same page as to what behaviour was expected in the workplace’.  Reports of positive experiences and outcomes when utilising the workshop material to respectfully challenge negative workplace behaviours.  Identified that the effect ‘teetered off’, and some suggested the need for ongoing education.  One informant stated that bullying-related training ‘never seems to work because we do it every year and nothing changes’. | | | **Confirmation**  Both data sets identify positive effects following the educational intervention (although not statistically significant).  **Expansion**  The qualitative data allowed participants to provide feedback and insight into the intervention’s effects at both a personal and a ward level.  **Discordance**  One informant from the qualitative strand did not feel that education improved workplace culture. |
