# Supplementary material for: Navigating Integration in Mixed‐Methods: A Practical Guide for Novice Nursing Researchers
Source: Res Nurs Health. 2025 Dec 20;49(2):193–201. doi: 10.1002/nur.70048 (PMC12954644; doi:10.1002/nur.70048)
Supplement: Supplementary file 3 — Supplementary Table 2: Initial Joint Display FINAL. [file NUR-49-193-s003.docx]

Supplementary Table 2: Initial Joint Display

| Findings | Quantitative Results  Survey Responses | Qualitative Results  Face to Face Interviews |
| --- | --- | --- |
| Exposure to negative acts | Across the total sample (n=130) 31% of participants (n=40) experienced bullying and 46% of participants (n=59) experienced incivility  There was no significant difference in nursing roles experiencing negative behaviours.  48% (n=12) participants identified managers as the main perpetrators of bullying in pre intervention survey  Registered nurse colleagues were identified as being the main source of bullying (73% (n=11)) and incivility (42%, n=15) in the pre-intervention and for incivility in the post-intervention (57%, n=13) | All informants in this study, at all occupational levels experienced negative workplace behaviours,  New staff to a ward were more likely to be the victims as perpetrators saw them as being vulnerable and weak  Informants described registered nurse colleagues and managers as being the perpetrators of negative workplace behaviours |
| Types of negative behaviours experienced | The most reported negative acts experienced by participants across all sites in both surveys were *work related bullying acts* and included being exposed to an unmanageable workload (75%, n=97), having opinions and views ignored (49%, n=64), being given tasks with unreasonable targets or deadlines (48% n=63, someone withholding information which affects your performance (44% n=57) | The behaviours that informants were exposed to in their workplace included unfair rostering and workloads, being made to work outside of their skill level, isolation and exclusion, information being withheld, being undermined, and contradicted in front of the team, and being singled out and made to look incompetent. |
| Types of positive behaviours experienced | No data on positive workplace practices | Informants spoke about instances of individual tiny acts of kindness and positive workplace practices such as pleasant introductions, orientation to the ward environment, knowing where the staff toilet and tearoom were located, and someone offering to be the person to help if needed  Social events, staff morning teas, holiday celebrations, or birthdays with cake all helped build camaraderie  The acceptance of learning as a lifelong process was also viewed as a crucial underpinning to positive workplace culture |
| Causes of negative workplace behaviours | No data | Informants described their tribes as having an *‘us vs them’* (Clinical nurse educator 1) attitude, where existing staff were described as territorial. Clinical nurse educator 6 described it as *‘being like a pack’*.  Nurses who did not cope and could not complete the expected workload faced negative workplace behaviours as a consequence.  Questioning practice or practicing in a different way than *‘what has always been done’* (Clinical nurse educator 5) led to bullying and uncivil behaviour  Informants identified that poor workplace conditions such as short staffing and heavy workloads had negative effects on nurse’s workplace interactions.  The ward was described as a reflection of the NUMS leadership  Nurse unit manager 3 described negative workplace behaviours as ‘a symptom of a struggling system |
| Implications of negative acts | 51% (n=21) of participants in the pre-intervention and 48% (n=12) of participants in the post-intervention indicated they had thought about leaving their current position due to negative behaviours experienced. | Negative behaviour was cited by informants as a reason that nurses leave the profession and had implications for recruitment.  One informant described it as a ‘*living hell’* (Nurse unit manager 1) and others shared feelings of embarrassment, fear, sadness, tiredness and feeling physically sick. They describe lacking self-confidence and the motivation to come to work, feeling burnt out and considering leaving, with new graduates questioning their career choice.  The behaviours impacted informants' home life with reports of increased irritability at home, increased alcohol consumption, and lack of sleep due to thinking about incidents that had occurred.  Affected the ward's culture and working environment.  Impacted patients’ quality of care |
| Nurses’ ways of coping | The most common ways of coping used by participants for both the control and intervention sites for pre- and post-intervention surveys included concentrating on what they had to do next (91%, n=113); Trying to analyse the problem to understand it better (90%, n=110), Turning to a work or substitute activity to take their mind off it (80%, n=98) and Talking to someone about how they were feeling (79%, n=93)  Across both the control and intervention sites, both before and after the intervention, Domain 1 - Problem-focused coping strategies were the most commonly used, followed by Domain 4 - Seeking social support. | Informants reported using various ways of coping, ranging from exercising, avoidance of the person/workplace, keeping work/life separate, giving up and acquiescing to fit in, or formulating a plan to deal with the negative behaviour  Informants also reported seeking support from others, including family, mentors, and also from Employee Assistance Programs. Although some informants reported taking sick leave or considered leaving their job,  Retaliation became a mechanism for coping. Informants reported that in response to negative behaviour experienced they began to withhold information, do the bare minimum, and refuse to help others |
| Impact of educational Intervention | There was a decrease in reported bullying in both the intervention group and control group, from 37% to 31% and from 29% to 21% respectively  There was a decrease in the number of participants who experienced incivility in the control group from 57% to 26%; however, the intervention group reported a rise from 44% to 50%  For the NAQ-R Domain 1 - Work related bullying exposure, the intervention group had a median score change of -2 (95%CI= -5.5 to 1.5) from the pre-intervention time period.  For the intervention group there were statistically significant decreases in the Ways of Coping Questionnaire Domain 4 – Seeking support (p=0.019) and Domain 6 – Self-blame (p=0.011) as coping strategies after exposure to the Respectful Workplace Workshops.  Improvement in the intervention groups' policy awareness from 82% (n=37) to 92% (n=33).  Improvement in intervention participants' perceived ability to challenge disrespectful behaviour, ability to use the resolution pathways, and knowing when to escalate to management, although not statistically significant  The intervention sites' intention to leave decreased from 56% (n=14) in the pre-survey to 47% (n=9) in the follow-up survey. | Informants described there being a ‘big push’ to attend the educational intervention and that staff initially had inspiration after first attending the workshops, as they were ‘all on the same page as to what behaviour was expected in the workplace’  Reports of positive experiences and outcomes when utilising the material from the workshops to respectfully challenge negative workplace behaviours.  Identified that the effect ‘teetered off’ and some suggested the need for ongoing education.  One informant stated that bullying related training “never seems to work, because we do it every year and nothing changes” |
